# Comparison of Heart Symptoms Aggravated by Motion

**Published:** 1885-07

**Authors:** 


### COMPARISON OF HEART SYMPTOMS AGGRAVATED BY MOTION.

ARSENICUM: Palpitation, with dyspnœa; worse when lying; less when moving.

ASPARAGUS: Violent palpitation from every motion.

AURUM: Violent beating of the heart, especially after exertion.

BOVISTA: Strong palpitation on going up-stairs and after exertion.

CALCAREA: Anxious palpitation from the slightest exercise.

COCCULUS: Palpitation on moving quickly.

DIGITALIS: Disease of the heart, with apnœa; from the slightest motion, much danger of suffocation, with yellow and blue face.

DIGITALIS: Fluttering at the heart after sudden and energetic motions, especially of the arms in an upward direction.

FERRUM: Palpitation, with fear; has to move about, can neither sit nor stand.

FERRUM: Palpitation; dyspnœa; fear; beats of the heart aggravated from the least motion.

PRUNUS SPINOSA: Furious beating of the heart, even when at rest, and great danger of suffocation from the slightest motion. Knocking at the heart, with labored breathing. Even from a very moderate motion the beats of the heart are fearfully aggravated. (CL. MULLER.)

SPIGELIA: Suffocative attack from motion; trembling feeling in the chest, on moving the arms, especially from moving the arms toward the head. (BESHMANN.) Cannot turn in bed without an attack of dyspnœa. (G. MAURO.) Motion aggravates the chest symptoms and causes faint feeling. (ELWERT.) After every rapid motion, palpitation. (LEHRŒN.)

STRAM.: From every motion, such violent palpitation that he cannot talk for hours. (HILBERGER.)

SULPHUR: Palpitation after going up-stairs or climbing mountains. Stitches in the sides of the chest, after vigorous bodily exercise. (NOAK.)

